# Transcription Factors in the Pathogenesis of Schizophrenia

**DOI:** 10.3390/life16050773

**Published:** 2026-05-05

**Authors:** Ahmed M. N. Helaly, Ahmed Al-Emam

**Affiliations:** 1 Forensic Medicine and Clinical Toxicology Department, Faculty of Medicine, Mansoura University, Mansoura 35516, Egypt; 2 Department of Pathology, College of Medicine, King Khalid University, Asir 61421, Saudi Arabia; alemam.ahmed@gmail.com

**Keywords:** schizophrenia, transcription factors, metabolic reprogramming, nuclear receptors, neurodevelopmental

## Abstract

Schizophrenia (SCZ) is a severe neuropsychiatric disorder characterized by a progressive clinical course and associated with a wide range of gene transcription signatures. This review examined studies retrieved from PubMed (published between 2005 and 2025) that investigated transcription factors (TFs) correlated with SCZ. Approximately 150 studies aligning with the eligibility criteria were selected. The synthesized evidence identified more than 40 TFs implicated in the pathogenesis and risk of SCZ. Based on functionality, these TFs were categorized into four groups: (1) progenitor cell TFs (TCF4, POU3F2, NKX2.1, EGR3), (2) stem cell TFs (MYC, SOX2, ASCL1, REST, NR2E1), (3) metabolic reprogramming TFs (HIF1, SREBPs, STATs, SOX9, NRF1, NRF2, p53, FOXO, ATF4, NF-κB), and (4) nuclear TFs (AhR, RXR). The discussion shed light on how these TFs in consort with hundreds of potential genes could shape the pathophysiology of SCZ. Indeed, SCZ represents a complex genomic, nuclear, metabolic, and immune disorder characterized by a diseased cellular microenvironment, with hypoxia emerging as a key feature. Although targeting TFs pharmacologically remains challenging, innovative therapeutic strategies—such as antineoplastic and antipsychotic agents that modulate the cellular microenvironment—may offer promising new directions for SCZ treatment.

## 1. Introduction

SCZ is a distressing neuropsychiatric disease affecting approximately 1% of the global population, with heritability confirmed by twin studies. The complex pathophysiology of SCZ involves hundreds or even thousands of genes [1], yet the mechanisms by which these genetic variants converge to produce the clinical phenotype remain incompletely understood.

TFs are master regulators of gene expression. These proteins bind to DNA near the gene sequence, while playing a key role in numerous signaling pathways involved in the pathogenesis of various diseases, including diabetes, inflammatory disorders, cardiovascular diseases, and cancers [2,3]. By moderating gene–environment interactions, TFs influence diverse biological activities such as cell cycle, metabolic processes, RNA polymerase activity, histone modifications, and cell development and differentiation. The disruption of these tightly regulated mechanisms, controlled by more than 1000 TFs, is therefore implicated in a wide range of diseases [4].

In the context of psychiatric illness, a broad array of TFs has been implicated in disease pathogenesis. Genome-wide association studies (GWAS) have demonstrated links between several TFs and SCZ [5], further showing that the genetic makeup of SCZ correlates with variations in TF binding sites rather than with the genes themselves [6]. Experimental work has also revealed somatic mutations at TF binding sites in post-mortem brains of individuals with SCZ; whole-genome sequencing identified CpG transversions and other mutations at TG sites overlapping chromatin-opening regions. Notably, these mutated DNA regions correlate with risk genes associated with neurodevelopmental disorders, especially during the perinatal period [7]. Complementing these findings, single-cell transcriptomics have significantly enhanced our understanding of neurogenesis, evolution of the cortex, and molecular interactions in various neuropsychiatric disorders [8,9].

Despite the clear involvement of TFs in SCZ, targeting them with small-molecule drugs remains challenging, as most TFs are considered “orphan” targets. Moreover, because TFs interact with multiple pathways, developing drugs with broad yet selective interaction profiles remains challenging. Although this represents a formidable obstacle, the successful identification of agents that modulate TF activity would provide decisive therapeutic tools [2,3].

Despite the growing body of evidence linking TFs to SCZ pathogenesis, no comprehensive synthesis has systematically categorized the diverse TF families involved or integrated their roles across developmental, metabolic, and nuclear signaling domains. The existing literature has largely focused on individual TFs or specific pathways, leaving a gap in our understanding of how distinct TF classes collectively contribute to the complex molecular landscape of SCZ. Addressing this gap is essential for identifying potential therapeutic targets and understanding the fundamental mechanisms driving disease progression.

This review aims to synthesize and categorize the major TF families implicated in the pathogenesis of SCCZ. Specifically, we discuss progenitor cell TFs (TCF4, POU3F2, NKX2.1, EGR3); stem cell TFs (MYC, SOX2, ASCL1, REST, NR2E1); metabolic reprogramming TFs (HIF1, SREBPs, STATs, SOX9, NRF1, NRF2, p53, FOXO, ATF4, NF-κB); and nuclear TFs (AhR, RXR), among others. By integrating findings from more than 40 TFs and hundreds of target genes, this review provides a conceptual framework for understanding SCZ as a complex disorder involving genomic instability, metabolic dysregulation, immune dysfunction, and a diseased cellular microenvironment with emerging hypoxia as a key pathological feature.

## 2. Methodology

### 2.1. Search Strategy and Information Sources

This systematic review was conducted following established methodological guidance for reviews. A systematic literature search was performed using PubMed and Web of Science databases. The search period extended from January 2005 to March 2025. The search strategy combined relevant keywords using Boolean operators. The primary search string comprised the following terms: (“schizophrenia” OR “psychosis” OR “psychiatric disorders”) AND (“transcription factors” OR “progenitor” OR “neurodevelopmental” OR “stem cells” OR “metabolic” OR “hypoxia” OR “nuclear factors” OR “inflammation”).

### 2.2. Eligibility Criteria

Studies were included if they met the following criteria: (1) peer-reviewed original research or review articles; (2) investigation of TFs in relation to SCZ; (3) human subjects or relevant animal models; (4) published in English; and (5) published within the 20-year timeframe (2005–2025). No restrictions were applied based on study design, provided the research offered clinical or experimental evidence relevant to TFs in SCZ pathogenesis.

### 2.3. Study Selection and Data Synthesis

The initial search yielded approximately 500 records. Titles and abstracts were screened for relevance, followed by a full-text review of eligible articles. Approximately 150 studies were incorporated into this review. Selection prioritized references with clinical and experimental evidence, followed by clinical data alone, and finally animal model studies. Where multiple sources addressed the same mechanism, preference was given to studies with clearly articulated hypotheses and robust methodological approaches. The synthesis was conducted narratively, and findings were organized based on the functionality of TFs.

### 2.4. Results and Discussion

This review synthesized evidence from 150 studies examining TFs in SCZ pathogenesis. Based on their functionality, TFs are categorized into progenitor cell TFs, stem cell TFs, metabolic reprogramming TFs, and nuclear receptors.

## 3. Progenitor Cell TFs

### 3.1. TCF4

Transcription Factor 4 (TCF4) is a progenitor cell TF belonging to the helix-loop-helix family. It has been implicated in the pathogenesis of neurodevelopmental disorders, including autism and SCZ, as well as other neuropsychiatric disorders [10].

Genetic evidence strongly links TCF4 to SCZ risk. Genome-wide association studies (GWAS) have identified rare copy number variants corresponding to the genetic predisposition of SCZ. A meta-analysis revealed that a single-nucleotide polymorphism (SNP) in intron 4 of TCF4 is associated with a high risk of SCZ, alongside several other indicators. Moreover, TCF4 collaborates with other TFs to modulate gene expression. TCF4 mutations have also been found in patients with Pitt–Hopkins syndrome, an autosomal-dominant disorder characterized by profound intellectual disability. Collectively, these findings indicate that TCF4 variations play a role in a variety of neuropsychiatric conditions like SCZ [11].

The mechanism by which TCF4 mutations affect the human brain remains incompletely understood. Studies using cultured neural progenitor cells (NPCs) derived from Pitt–Hopkins syndrome patients have revealed reduced proliferation with poor differentiation towards neurons. Knockout of TCF4 results in decreased Wnt signaling and subsequent downregulation of SOX gene expression. These in vitro studies also demonstrated defective cortical neuron bulk and disrupted electrical activity in mutation-developed organoids. Importantly, restoration of TCF4 function improved the neuronal phenotype, suggesting that TCF4-related Wnt signaling represents a potential therapeutic target for SCZ [12].

Animal models have corroborated these findings. The Tcf4+/tr mouse model exhibits decreased expression of parvalbumin, vasoactive intestinal peptide (VIP+), and cortistatin (CST+) interneurons in the cortex, along with a corresponding increase in cholinergic interneurons in the striatum [10]. Transgenic mice overexpressing murine TCF4 display cognitive decline and sensorimotor gating deficits that parallel SCZ in humans [13]. Conversely, TCF4 loss-of-function mutations induce Pitt–Hopkins syndrome, a rare neurodevelopmental disorder associated with intellectual disability, delayed developmental milestones, and autistic features. Partial or complete knockout of TCF4 leads to reduced dendritic arborization in both the frontal lobe and hippocampal area. These findings underscore the importance of TCF4 in synaptic plasticity and neuronal maturation in neurodevelopmental diseases such as autism and SCZ; however, the role of TCF4 in autism is beyond the scope of this review [14].

### 3.2. POU3F2

POU3F2, also known as a brain-specific transcription factor, has been recognized as a potential contributor to the pathogenesis of SCZ. However, the mechanistic details of this TF in SCZ remain incompletely understood [15].

POU3F2 modulates a group of SCZ-related genes, as confirmed by gene knockdown and RNA-sequencing studies of the human NPC transcriptome. One such target is TRIM8 (tripartite motif containing 8), which is abnormally transcribed and expressed in patients with SCZ. Experimental evidence has demonstrated that POU3F2-related TRIM8 expression is increased through binding to the SCZ-associated SNP; rs5011218. This variant disrupts POU3F2 binding efficacy at the promoter site of TRIM8 [15].

Both POU3F2 and TRIM8 co-modulate multiple pathways related to neural maturation and synaptic activity. Blockade of either POU3F2 or TRIM8 promotes the propagation of undifferentiated progenitor cells while simultaneously hindering their differentiation. This results in defective excitatory synaptic transmission, inhibited neuronal differentiation, and impaired synaptic transmission activity of NPC-derived neurons. The SCZ-associated SNP rs5011218 thus indicates that POU3F2-regulated TRIM8 expression is implicated in the pathophysiology of SCZ by modulating neural growth and synaptic propagation [15].

Beyond its role in SCZ, POU3F2 targets neural stem cells and modulates hundreds of genes, playing an important role in both neurodegenerative and psychiatric disorders [16].

### 3.3. NK2 Homeobox 1 (NKX2.1)

NKX2.1 plays a particular role in the development of the schizophrenic brain. NKX2.1 is selectively expressed from the perinatal period through adolescence, a developmental window critical for psychosis risk, in multiple tissues including the brain, thyroid gland, parathyroid gland, lungs, skin, and enteric ganglia. Additionally, NKX2.1 serves vital functions at the interface of the brain, the endocrine system, and the immune system [17].

During brain development, NKX2.1 is expressed in NPCs as they differentiate into specific cell populations, including GABAergic and cholinergic neurons, astrocytes, and oligodendrocytes in distinct regions of the frontal lobe. Mature limbic circuits show high expression of NKX2.1, which is associated with purposive behavioral response, social communication, reproduction, fear reactions, light response, and other homeostatic mechanisms [17].

Beyond its central nervous system functions, NKX2.1 is essential for the development and maturation of the thyroid gland and the respiratory organs. It is also implicated in calcium dynamics and immune reactions. Furthermore, NKX2.1 interacts with a battery of genes known to be responsible for SCZ, suggesting a broad pathogenic role [17].

### 3.4. EGR3 (Early Growth Response 3)

The EGR gene family, is a subset of immediate early genes (IEGs) encoding TFs that are expressed in response to environmental stressors in the brain [18]. The EGR gene family orchestrates numerous functions in the neural cells, including synaptic plasticity, and is considered a component of SCZ pathogenesis [19].

EGR3 is a master regulator of a battery of genes involved in the risk of SCZ as well as bipolar disorder. Moreover, it is implicated in the pathogenesis of neurodegenerative disorders [20]. Through high-throughput screening and bioinformatics, EGR3 has emerged as a key TF required for neuronal activity in the mouse hippocampus. GWAS have shown that impaired EGR3 correlates with SCZ risk. Furthermore, EGR3 promotes a transcriptomic signature that enables interaction with environmental stressors by modulating behavior, memory, and synaptic plasticity [21]. These genes and mechanisms may relate to the pathology observed during adolescence [22].

MicroRNAs (miRNAs) and TFs regulate gene expression in a coordinated manner, playing a role in gene expression in SCZ. EGR3 and has-miR-195 have been demonstrated to regulate signaling pathways in the brain. Polymorphisms in both TFs and miRNA may contribute to the etiology of SCZ. They create what is known as a feed-forward loop to regulate the transcriptome in diseases, including SCZ [23].

Animal models have provided further insights. EGR3 knock-out mice exhibit SCZ-like manifestations that respond to antipsychotic treatment. These mice slept for shorter periods, highlighting the importance of EGR3 in sleep regulation in psychiatric disorders [24].

Gene–environment interaction studies have demonstrated that synaptic plasticity in response to different stressors contributes to the development of SCZ. Polymorphisms of EGR3 and its target protein ARC (activity-regulated cytoskeleton-associated protein) confer risk for developing SCZ in Americans of European descent as well as African Americans. ARC SNPs have also been identified as a risk factor in Chinese populations. Carriers of rs35900184 are more susceptible to SCZ. The application of next-generation sequencing may be utilized worldwide to discover additional correlations [25].

### 3.5. Interaction Between Neurodevelopmental Factors

While each progenitor cell TF discussed above has distinct roles, emerging evidence suggests functional interactions among them during neurodevelopment; however, the literature focusing on this interaction remains scarce.

NKX2. 1 interacts with POU3F2 during the development of the mammalian hypothalamus. However, NKX2.1 targets NPCs in the early stages of progenitor cell activity, whereas POU3F2 targets neurons in the post-mitotic phase. This temporal distinction suggests that these factors may interact in a sequential rather than parallel manner [26].

A second example involves TCF4 and NKX2.1 in olfactory bulb development. Normal olfactory bulb development requires a normal population of oligodendrocytes to process the olfactory functions. TCF4 is crucial in mediating normal cell death, thereby maintaining the normal number of NKX2.1-positive oligodendrocyte progenitor cells located in the medial ganglionic eminence [27]. Notably, reduced olfactory bulbs have been found in individuals with SCZ as well as in those with depression and anxiety [28], with similar findings reported in animal models of SCZ [28,29].

Collectively, these interaction studies, though scarce, indicate that progenitor cell TFs do not operate in isolation. Their coordinated, temporally regulated activities during critical developmental windows may converge on common pathways underlying SCZ risk. In addition to the TFs discussed above, several other progenitor-associated TFs have been implicated in SCZ, including NPAS3, NPAS4, Lhx6, and NEROG1.

## 4. Stem Cell TFs and SCZ

Stem cells play a complex role in the pathogenesis of SCZ, though this area remains underexplored and requires further investigation. Most experimental research on stem cells in SCZ has focused on induced pluripotent stem cell (iPSC) models of psychosis [30]. The application of stem cells in SCZ treatment represents a relatively new approach in psychosis management; however, to our knowledge, no stem-cell-based therapy has been approved by the FDA for this indication [31]. Nevertheless, promising evidence suggests that stem cells can regenerate damaged neurons, and neural stem cells have the capacity to modulate neural plasticity and improve cognitive function in various neuropsychiatric disorders [32].

A unique study demonstrated that electroconvulsive therapy (ECT) for SCZ and depression increased the expression of stem cell TFs, including OCT4, SOX2, cMYC, and KLF4. This finding suggests that stem cells are involved in neural plasticity and the Wnt signaling pathway, positioning both as potential therapeutic targets for SCZ [33].

### 4.1. MYC TF and SCZ

The MYC family comprises a group of transcriptional regulators that play substantial roles in mammalian development, including stem cell maintenance and regeneration. MYC has the capacity to activate pathways that stimulate cell growth and proliferation and is commonly overexpressed in various tumors, including brain tumors [34].

In the context of neuropsychiatric disease, MYC is a TF and oncogene implicated in numerous conditions, including neurodegenerative disorders like Alzheimer’s disease and SCZ. MYC exerts its regulatory functions through long non-coding RNAs and microRNAs, which serve as second messengers. One such long non-coding RNA, MINCR, is dysregulated in SCZ as well as other disorders [35].

The competing endogenous RNA (ceRNA) hypothesis offers a framework for understanding the interaction between coding and non-coding RNAs, potentially explaining how MYC contributes to the complex regulatory transcriptome signature observed in SCZ [36].

**Figure 1:**
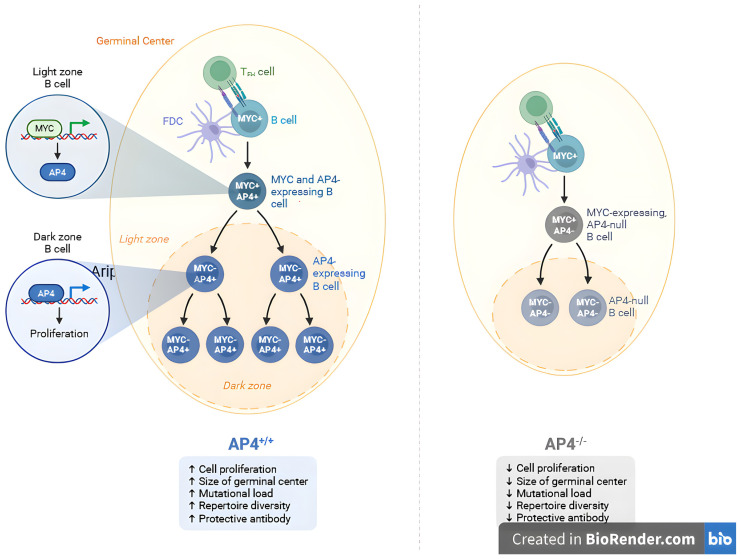
Schematic representation of MYC TF signaling. MYC binds specific DNA motifs to regulate a broad network of pathways involved in cellular proliferation, immune modulation, and neuropathology.

Figure 1 illustrates the MYC signaling pathway as a model for understanding how TFs modulate immune system function, affect multiple organs, and activate gene networks to induce diverse cellular responses.

### 4.2. SOX2 (Sex-Determining Region Y-Box 2) Factor and SCZ

While MYC primarily regulates cellular proliferation, the transcription factor SOX2 is critical for maintaining stem cell identity and neural development. SOX2 is a TF with conserved function across vertebrates. It is essential for the development of the central nervous system from the earliest embryonic stages [37].

Human SOX2 mutation causes a range of central nervous system (CNS) defects, including ocular and hippocampus dysfunctions, cognitive disability, and motor control impairment. Similarly, SOX2 knockout mice expressed very wide phenotypic changes in both developing and postnatal neural tissue. The developing neural tube and regions such as the hippocampus and frontal lobe are more susceptible to SOX2 disruption than other brain areas. SOX2 is critical for neural precursors, GABAergic interneurons, glutamatergic projection neurons, and Bergmann glia. Human SOX2 mutations have been linked to a genetic profile associated with neurodevelopmental disorders [37].

Beyond its role in neural development, SOX2 regulates the stemness of embryonic stem cells. The human SOX2 overlapping transcript (SOX2-OT) DNA binding site contains multiple exons with numerous transcription start sites, generating hundreds of transcript variants. This long non-coding RNA, SOX2-OT, is involved in the pathogenesis of SCZ and anorexia nervosa [38].

### 4.3. Achaete-Scute Homolog 1 (ASCL1)

ASCL1 is a basic-helix-loop-helix (bHLH) TF that works in combination with OLIG2 to regulate neurogenesis. Single-cell RNA sequencing (scRNA-seq) has revealed a transcriptomic signature indicating that ASCL1 plays a key role in migratory neural stem cell (NSC) and astrocyte-like tumor cell types. Additionally, ASCL1 is implicated in the upregulation of ribosomal protein expression, oxidative phosphorylation programming, metastatic tumors, and drug resistance genes [39].

ASCL1-dependent cellular and molecular programs have been linked to the pathophysiology of SCZ. ASCL1-knockout cell lines display a transcriptomic signature that converges with molecular pathways underlying the pathogenesis of SCZ and bipolar disorder. ASCL1 promotes cell mitosis, neuronal projection, and neuropeptide signaling and facilitates cell–cell contacts, including the maturation of the synaptic network [5].

The primary role of ASCL1 is during development; however, its function in the mature brain is restricted to fewer gene expressions related to neuroplasticity [5]. ASCL1 interacts with its co-partner PHOX2B (paired-like homeobox 2B) to regulate catecholamine-synthesizing enzymes. Both factors have been associated with the pathogenesis of bipolar affective disorder and Parkinson’s disease (PD), where catecholaminergic dysfunction is a common mechanism [40].

### 4.4. Repressor Element-1 Silencing TF/(REST)

REST is also known as neuron-restrictive silencing factor (NRSF), a key regulator of epigenetic neuronal gene expression. REST targets a large number of genes in the brain, indicating its extensive involvement in maintaining normal nervous system function. Furthermore, REST protects the nervous system against various injuries and stressors [41].

At the molecular level, nuclear translocation is essential for REST to function properly. Recent research has revealed that REST nuclear mislocalization or dysfunction is implicated in numerous neurological diseases. However, the wide range of variation in normal REST expression across different brain cell types complicates the interpretation of REST expression levels in various brain diseases [41].

REST functions as a key regulator across the entire lifespan of the nervous system, supporting embryonic brain development, guiding neurogenesis, sustaining adult neurogenic processes, and helping the aging brain adapt to physiological and environmental stressors [41].

Neuroimaging studies have provided insights into REST’s role in psychosis. Positron emission tomography (PET) and magnetic resonance imaging (MRI) perfusion studies of patients with active psychosis have demonstrated hyperperfusion of the hippocampus. This activity reflects hyperactive NMDA receptors in the hippocampal CA3 region. These findings suggest that REST activity is reduced during active psychotic states. REST is implicated in the maturation of NMDA receptors through epigenetic mechanisms. The hyperexcitable hippocampus may contribute to false memories and delusions in SCZ [42].

### 4.5. Nuclear Factor 2E1 (NR2E1)

NR2E1 is located at the 6q21-22 chromosomal locus, a region previously associated with bipolar disorder and SCZ. NR2E1 knock-out mice exhibit defective neurogenesis, cortical and limbic abnormalities, aggression, hyperexcitability, and cognitive impairment. These findings position NR2E1 as a key factor in mental disorders. Studies on NR2E1 messenger RNA (mRNA) expression have revealed a specific signature in the human forebrain that correlates with psychiatric disorders, including SCZ and mood disorders [43].

## 5. Metabolic Reprogramming TFs in SCZ

Having discussed TFs involved in stem cell maintenance and neurodevelopment, this section examines TFs that regulate metabolic reprogramming, which is a critical but underappreciated aspect of SCZ pathogenesis.

Multiple TFs modulate cellular metabolism, including MYC, HIF1α, SREBPs, and POU1F1, STATs, SOX9, NRF1, NRF2, and PBX3. Among these, MYC has already been discussed in the stem cell transcription factor section. No data was available about PBX3 and POU1F1 in SCZ.

Some TFs discussed in this section, including STATs, p53, and NF-kB, have primary roles in immune signaling and stress responses but also regulate metabolic pathways relevant to SCZ pathogenesis.

### 5.1. HIF1 (Hypoxia-Inducing Factor-1)

HIF-1 consists of two subunits: HIF-1α and HIF-1β proteins. HIF-1 is upregulated in response to hypoxia and binds to its DNA motifs, stimulating the transcription of hypoxia-inducible genes. Thus, HIF-1 supports cellular adaptation to hypoxic stress [44].

- Hypoxia and SCZ

Numerous prenatal and perinatal injuries in children have been associated with the development of psychosis. These include prenatal infection and difficult labor, both of which imply a degree of hypoxia [45]. Perinatal stressors, including obstetric complications, induce psychosis in a small number of cases, suggesting gene–environment interaction [46]. Prenatal hypoxic individuals who develop psychosis show reduced left amygdala volume on MRI examination [46,47].

Experimental research supports these human clinical findings. Animal models of SCZ involving hypoxia show reduced HIF1 and increased vascular endothelial growth factor (VEGF) expression [48]. Additionally, maternal smoking during pregnancy induces fetal hypoxia, increasing the risk of SCZ in offspring, with an increased incidence of developing negative symptoms. Paternal smoking does not show a significant correlation [49].

B. Hypoxia in the adult brain and SCZ

The expression of hypoxia-related genes differs between patients with SCZ and healthy controls [50]. Low birth weight and hypoxia have been identified as risk factors for developing SCZ later in life. These findings support the view that genetic susceptibility to SCZ increases the vulnerability of the developing brain to adverse effects arising from obstetric complications. Epigenetic mechanisms may play a role in this process [51].

C. Hypoxic-inflammatory model of SCZ

Macrophage migration inhibitory factor (MIF) is a cytokine with multiple functions, including promoting neurogenesis and neuron protection. Astrocytes are the primary site of MIF expression in the brain. Research indicates that hypoxia acts as a risk factor for SCZ by inducing MIF expression through HIF binding, which in turn activates the transcription of genes containing hypoxia-response elements (HREs). An SNP in the MIF promoter (rs1700403) has been linked to SCZ via inhibition of the hypoxia-related HIF pathway-induced MIF expression [52].

D. HIF and long non-coding RNA

A recent study showed that numerous long non-coding RNAs (lncRNAs) are linked to SCZ through HIF-1 signaling and oxidative phosphorylation pathways. The pathogenesis of SCZ has been associated with disease-related alterations in the expression of lncRNA-binding protein (RBP-lncRNA) complexes and their interaction networks, offering new insights into underlying molecular mechanisms and potential biomarkers for the disorder [53].

E. Genes driven by hypoxia

Hypoxic stress during embryonic development modulates neuronal genes via HIF-1 secretion. Intrinsic hypoxia appears to be a core mechanism contributing to SCZ pathogenesis. The hypoxia response affects a range of genes, including AKT1, BDNF, CAPON, CCKAR, CHRNA7, CNR1, COMT, DNTBP1, GAD1, GRM3, IL10, MLC1, NOTCH4, NRG1, NR4A2/NURR1, PRODH, RELN, RGS4, RTN4/NOGO, and TNF [54]. Further research is needed to explore the pivotal role of hypoxia in SCZ, both during development and in adulthood.

### 5.2. SREBPs (Sterol Regulatory Element Binding Proteins)

SREBPs are a group of TFs that regulate cholesterol metabolism. They have been extensively studied for their roles in brain cholesterol metabolism, amyloid turnover, and tau tangles synthesis. SREBPs maintain cholesterol homeostasis in the brain [55]. There are three subtypes of SREBPs:

- SREBP-1a increases the gene transcriptions related to cholesterol and fatty acid production.
- SREBP-1c promotes adipogenesis.
- SREBP-2 regulates cholesterol synthase and low-density lipoprotein (LDL) receptors. Additionally, SREBP-2 compensates for cholesterol depletion by upregulating cholesterol production and absorption [55].

Research has demonstrated a relationship between myelin dysfunction and SCZ. Oligodendrocytes are highly fortified with lipids, including cholesterol, phospholipids, and glycosphingolipids. Consequently, stable lipid metabolism is essential for maintaining the integrity of the myelin sheath. SREBP1 and SREBP2 have been implicated as genetic risk factors for SCZ, consistent with evidence that defective myelination is a core component of SCZ pathogenesis [56].

Animal models have provided further insights. SREBP-1c knockout mice exhibit SCZ-like behavior. Structural examination revealed increased lateral ventricle volume in these knockout mice. The expression of several γ-aminobutyric acid (GABA) receptor subtypes and/or glutamic acid decarboxylase decreased in brain regions such as the hippocampus and the medial prefrontal cortex. Thus, SREBP-1c deficiency has been linked to the lateral ventricle enlargement observed in individuals at risk for SCZ, as well as to behavioral abnormalities associated with impaired GABAergic neurotransmission [57].

**Figure 2:**
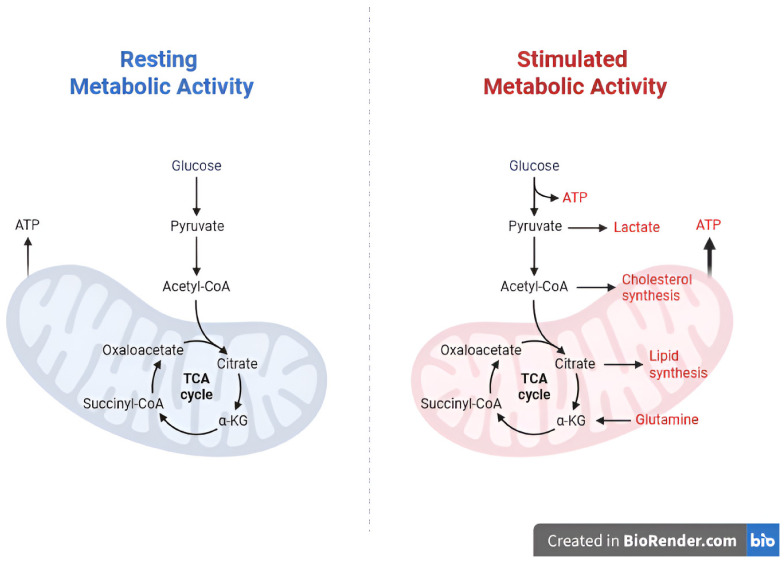
Schematic representation of the tricarboxylic acid (TCA) cycle as a central metabolic hub. The TCA cycle integrates cellular energy demands with hypoxia, cholesterol metabolism, oxidative stress, and subsequent inflammatory responses in the context of SCZ pathogenesis.

Figure 2 illustrates the Tricarboxylic Acid Cycle (TCA cycle) as a central hub linking cellular energy demands with hypoxia, cholesterol metabolism, oxidative stress, and subsequent inflammatory response.

### 5.3. STATs (Signal Transducers and Activators of Transcription)

STATs are key components of the Janus kinase (JAK)-STAT signaling pathway, a transmembrane signaling system that enables cellular responses to external stimuli. This pathway is controlled by a wide range of ligands, including cytokines, interferons, growth factors, and other stimuli, that activate JAK–STAT signaling under both physiological and pathological conditions. The resulting downstream responses encompass cellular proliferation, metabolic regulation, immune activation, inflammation, and malignant transformation [58].

Metabolic adaptation is essential for the T cells to respond to their functional demands, including movement and differentiation in response to various stressors. The JAK-STAT pathway is a canonical regulator of T cells’ adaptation to immune memory through both direct and indirect mechanisms. Indirect effects include upstream modulation of cytokines and other TFs, positioning JAK-STAT as a key regulator of T cell metabolism [59].

The link between inflammation and psychosis is well established. Activated inflammatory cells secrete cytokines that stimulate the JAK-STAT1 pathway, promoting neuropsychiatric responses in various mental disorders [60]. Moreover, the interferon (IFN)-induced STAT1 signaling pathway is a canonical immune pathway that has also been implicated in regulating neuronal activity. This pathway is augmented in the brains of patients with autism spectrum disorder (ASD) and SCZ [61].

Animal models have provided causal evidence. Overactivation of STAT1 elicits pathological responses in mice that closely mirror SCZ-related neurobehavioral abnormalities. In this STAT1 gain-of-function mouse model, mice exhibited heightened excitability alongside neural hypoactivity, accompanied by reduced neuronal counts within the caudate–putamen. Together, these findings point to a convergence of aberrant STAT1 signaling, striatal vulnerability, and circuit-level dysfunction that may underlie key features of SCZ-associated pathology. It has been suggested that STAT-dependent transcriptional programs in neurons may underlie these behavioral phenotypes across neurodevelopmental disorders [61].

Genetic evidence further supports STAT involvement in SCZ. SNP rs1344706 is located in the non-coding region of the ZNF804A gene and is associated with lower STAT expression in fetal brains. This genetic profile corresponds to a high risk of SCZ and mood disorders. The molecular and cellular mechanisms of ZNF804A are not well characterized. Two-hybrid and bioluminescence-based yeast assays generated a high-confidence protein–protein interaction (PPI) network for ZNF804A, identifying 15 proteins linked to SCZ pathology. One of these proteins is STAT2, an interferon-targeted TF. Further experimental details revealed that STAT2 interacts with the ZNF804A N-terminus and interferon treatment in HEK293 cells co-translocates both proteins from the cytoplasm into the nucleus. Furthermore, STAT2 overactivation was experimentally observed in cell lines using a luciferase reporter [62].

Additional links between SCZ and cancer have emerged through STAT signaling. The antipsychotic drug sertindole has anti-bladder cancer activity by inducing apoptosis. This action is mediated by reversing the anti-apoptotic STAT3/BCL-xL transcription activity. These findings highlight common mechanisms of cross-talk between SCZ and cancer [63].

Human studies on patients with SCZ and their twins have revealed atypical methylation levels of FYN, STAT3, RAC1, and NR4A2, further implicating epigenetic regulation of STAT-related pathways in SCZ [64].

Immune dysfunction has been confirmed as a molecular finding in psychotic disorders such as SCZ and bipolar disorder with psychotic features. Dysfunctional JAK-STAT signaling has been proposed to explain the role of inflammation in neurodevelopmental disease pathogenesis. Neurodevelopment and the immune system may jointly contribute to the pathology of SCZ and cognitive disability [64,65].

### 5.4. SOX9 (SRY-Related High-Mobility-Group Box 9)

SOX9 is a TF that controls cell fate types in multiple vertebrate tissues. Its gene locus is large and complex, containing various tissue-specific enhancers. SOX9 is regulated by numerous enhancers that influence chondrocytes, Sertoli cells, and cranial neural crest cells [66].

Embryonic cell maturation and differentiation require metabolic reprogramming to activate the transcriptional machinery necessary for modulating gene expression. However, the interaction between gene expression and metabolism is not fully understood. Studies have found that glycolysis modulates histone lactylation in embryonic cells such as neural crest cells. SOX9 plays a role in this process by facilitating histone modification with lactyl-CoA. Thus, SOX9 promotes metabolism-induced epigenetic control of neural crest cells [67].

Human and animal cell lines have demonstrated that SOX9 expression is largely restricted to astrocytes, except for ependymal cells. NPCs also express SOX9, and reactive astrocytes continue to express SOX9 during adulthood and aging [68].

SOX9, together with factor1-A and Notch 2, is responsible for astrocyte maturation. Defective astrocyte differentiation due to genetic predisposition leads to impaired neuronal matrix and disrupted signaling. Consequently, glutamate and dopamine signaling become dysregulated. Astrocytes are thought to contribute to the neurodegenerative component of SCZ that develops later in life [69].

Post-mortem brain analyses have identified SOX9, along with several immune-related proteins including LC1A3, AQP4, GJA1, ALDH1L1, SLC4A4, EGR1, NOTCH2, PVALB, ID4, ABCG2, METTL7A, ARC, F3, and EMX2 as promising biomarkers of SCZ. Bioinformatics studies further support the utility of SOX9 as a potential early diagnostic indicator for SCZ [70].

### 5.5. NRF1 (Nuclear Respiratory Factor 1)

NRF1 acts as a sensor of proteasome deficiency by stimulating gene transcription to compensate for insufficient proteasome activity. This TF plays a conserved role across different organisms, from yeast to humans. Additionally, NRF1 functions as an endoplasmic reticulum (ER) stress response factor. It can be translocated retrograde to the cytosol, targeting the ER-associated degradation pathway (ERAD). The NRF1-proteosome axis could be a target for drugs treating neurodegenerative disorders [71] and possibly SCZ, given that NRF1 is implicated in its pathogenesis [72].

NRF1 has been shown to regulate the expression of proteins involved in oxidative phosphorylation and mitochondrial energy homeostasis [72]. Human studies indicate that the rs1399178 variant modulates SCZ risk by altering NRF1 binding affinity, thereby reshaping the transcriptional landscape of its downstream targets [73].

Post-mortem brain analyses further suggest that NRF-1 serves as a key upstream regulator of PGC-1α and several SCZ-relevant genes, including PVALB, Syt2, and Cplx1, implicating it as a valuable prognostic marker in disease pathogenesis. These findings point to a disruption of PGC-1α- and NRF-1-dependent transcriptional programs within the cortex of individuals with SCZ. Consequently, therapeutic strategies aimed at enhancing PGC-1α activity or modulating NRF-1-driven transcription may offer a rational approach for targeting multiple dysregulated gene networks and improving cognitive function in SCZ [74].

The function of NRF1 may be influenced by CpG methylation. This epigenetic mechanism regulates SCZ signaling. NRF1 methylation is also associated with TNIK (encoding TRAF2- and NCK-interacting kinase) gene methylation. Further research is needed to track the methylation of NRF1 and the related genes in relation to SCZ treatment [75].

Experimental work has indicated that sirtuin modulation inhibits apoptosis by increasing histone 3 acetylation. This mechanism rescues mitochondrial membrane potential loss, modifies mitochondrial calcium buffering capacity, reduces superoxide radical levels, and increases the expression of metabolic modulators (NRF11 and NFE2L2). These results contribute to understanding the hypoxia response during prenatal development and pregnancy-related pre-eclampsia [76].

### 5.6. NRF2 (Nuclear Factor Erythroid 2-Related Factor 2)

NRF2 is a TF that serves as a key defense pathway against oxidative stress and inflammation, both of which are implicated in many neuro-psychiatric disorders, including SCZ. NRF2 plays an essential role in modulating ferroptosis, a form of regulated cell death driven by iron-dependent lipid peroxidation, in a wide range of neurological diseases. The aging process induces a decline in NRF2 expression and its related genes (HO-1, Nqo-1, and Trx), coinciding with increased cell death-related disorders [77].

Redox dysfunction is a key mechanism promoting the development of psychosis. Oxidative stress and decreased antioxidants are markers of cellular dysfunction in SCZ, particularly during active psychosis. Redox stability is disrupted in the medial prefrontal cortex, striatum, and thalamus, as evidenced by altered levels of glutathione, a key marker of cellular redox balance. Disruption of redox homeostasis is associated with reductions in parvalbumin-expressing GABAergic interneurons and the loss of their surrounding perineuronal nets, alongside white matter abnormalities and microglial activation [78].

The KEAP1 (Kelch-like ECH-associated protein 1) pathway is a key regulator of NRF2 activity and represents a promising target for restoring redox balance. Redox control of molecular pathways involved in SCZ may offer a therapeutic strategy for treating psychosis [78].

Experimental studies have established a critical role for NRF2 in regulating cellular energy metabolism and maintaining mitochondrial stability. NRF2 is also implicated in metabolic reprogramming in cancer. Further investigation is needed to determine how NRF2-dependent metabolic reprogramming manifests in psychiatric disorders and to identify potential therapeutic entry points [79].

### 5.7. p53 (Tumor Protein p53)

p53 is a well-characterized TF best known for its role as a tumor suppressor. Proteomic data from schizophrenic NPCs have demonstrated altered expression of DNA damage response proteins, cell cycle regulators, and p53 target genes during early cell development into the adult brain [80].

The low incidence of cancer observed among individuals with SCZ may be related to the p53 activity. It has been proposed that heightened p53-mediated apoptosis contributes to the development of SCZ and may explain the reduced tumorigenesis observed in affected individuals [81].

Recent studies have identified additional roles of p53 beyond tumor suppression, including its function in metabolic homeostasis. p53 interacts with various metabolic pathways to maintain metabolic adaptation to cellular stress. Furthermore, rescue of mutant p53 proteins has been shown to initiate metabolic reprogramming in cancer cells, thereby supporting cancer progression. However, the potential role of gain-of-function p53 mutations in psychosis requires further investigation [82].

p53 also participates in adult neurogenesis through mechanisms such as epigenetic regulation of neurogenesis in the subgranular zone of the hippocampus [83].

### 5.8. FOXO TF (Fork Head Box O)

The FOXO family is a group of TFs involved in various physiological and pathological conditions, including cellular homeostasis, stem cell viability, cancer, metabolic disorders, and cardiovascular diseases. Genetic studies have reported that the FOXO family plays an evident role in the lifespan extension across many species. Furthermore, scientific evidence demonstrates that FOXO3 is the second most transcribed gene associated with a powerful human hormesis response, making it a promising target for treating aging-related diseases [84].

The mammalian FOXO family consists of FOXO1, FOXO3, FOXO4, and FOXO6, which correspond to DAF-16 in *Caenorhabditis elegans*. DAF-16 induces longevity in animals, indicating that FOXO TFs promoted stress resistance by activating stress response pathways. These TFs modulate redox status, thereby showing the accelerated aging process, and are implicated in many diseases such as cancer and diabetes [85].

The incidence of psychiatric diseases increases with aging, and the epigenetic clock correlates with psychosis development and higher mortality rates. Lithium, a well-established drug used to treat bipolar disorder with psychotic features, often as an adjunctive treatment for psychosis, requires β-arrestin and SGK (glucocorticoid-inducible kinase) to inhibit the nuclear localization of DAF-16 in *C. elegans* [86].

Inhibition of DAF-16 does not imply a negative association with SCZ pathogenesis. The improved stress resistance may explain the decrease in DAF-16 in *C. elegans* in response to the antipsychotic treatment. Studies have demonstrated that FOXO pathway-related genes are altered in patients with SCZ, and this modified transcriptomic signature may be due to olanzapine treatment or the duration of the disease [87].

### 5.9. Activating TF 4 (ATF4)

ATF4 is considered a vital regulator of learning and memory. Reduced hippocampal ATF4 expression has been reported to induce defective synaptic plasticity, impaired memory, and disrupted glutamatergic activity [88].

Mutations in the DISC1 gene (disrupted in schizophrenia 1) lead to loss of interaction with ATF4, resulting in defective cellular trafficking in SCZ [89]. Additionally, an ATF4 SNP has been linked to SCZ risk in Polish populations, warranting larger-scale studies of ATF4′s role in SCZ [90].

High-resolution atomic structure analysis of the DISC1-ATF4 complex has revealed that mutated DISC1 disrupts normal DISC1-ATF4 interaction, leading to excessive ATF4 binding to DNA targets and aberrant gene transcription. Furthermore, iPSC models of SCZ with defective DISC1-ATF4 interaction exhibit gene expression patterns reflecting synaptic abnormalities [91].

Single-cell RNA sequencing has demonstrated the role of ATF4 in regulating amino acid metabolism in lung fibroblasts. Interestingly, the mechanistic target of rapamycin (mTOR) pathway is a downstream signaling cascade of ATF4 that regulates the tricarboxylic acid cycle (TCA) cycle metabolism. These findings have been revealed in diseases such as idiopathic pulmonary fibrosis [92].

### 5.10. Nuclear Factor-kappaB (NF-κB)

NF-κB is a master TF that controls the immune responses to various stimuli. Activation of NF-κB in response to different stressors inhibits neuronal apoptosis and promotes neuroplasticity. Evidence from in vivo and in vitro studies has demonstrated that NF-κB is a target of many cytokines, neurotrophic factors, oxidative stress signals, and intracellular calcium [93].

Dysregulated NF-κB signaling contributes to neuroinflammation, a key component of psychosis pathology. The cortical microenvironment in individuals with SCZ exhibits evidence of defective immune responses, consistent with dysregulation of upstream TFs that orchestrate neuroimmune signaling. However, the causes of these cascades remain poorly understood [94].

Inflammatory cytokines and NF-κB have been measured in blood samples from patients with bipolar disorder and major depression. These studies demonstrated increased NF-κB expression in peripheral blood mononuclear cells, monocytes, and lymphocytes [95].

Experimental work has investigated shared pathogenic mechanisms between SCZ and mood disorders, as well as possible comorbidities such as diabetes. The data indicate that these conditions share common inflammation-related genes. Signaling pathways, including TNF signaling, IL-17 signaling, and NF-kappa B signaling, are involved in the integrity of dopaminergic and GABAergic synapses, suggesting potential common pathways in SCZ and depression [96].

Genetic studies have provided additional evidence. An Italian study revealed that GRIN1 SNP G1001C affects NF-kappa B transcription and is associated with increased risk of SCZ [97].

## 6. Nuclear Factors and SCZ

The TFs discussed in this section are characterized by their nuclear localization and direct binding to DNA response elements as receptors for small-molecule ligands. Many of these belong to the nuclear receptor superfamily and function as ligand-activated TFs.

Among these, the aryl hydrocarbon receptor (AhR) and retinoid X receptor (RXR) have been most extensively studied in the context of SCZ and are discussed in detail below. Additional nuclear receptors implicated in SCZ are summarized in Table 1.

### 6.1. AhR

AhR is a cytosolic TF. Dioxin is a full agonist of AhR [98]. AhR is activated by various endo- and exogenous ligands, which, upon activation, translocate it to the nucleus, bind to its DNA recognition motif, and induce transcription of target genes. In the cytosol, AhR remains inactive and is bound to heat shock protein 90 HSP90 [99].

AhR is differentially expressed in the gastrointestinal tract (GIT) and the blood–brain barrier. It interacts with diverse endogenous and exogenous ligands, including dopamine, thereby modulating the gut–brain axis. AhR-mediated cross-talk among the neuroamine, microbiome, and immune system has been implicated in SCZ pathogenesis [100].

Proteomic studies of hippocampal tissue from patients with SCZ and bipolar disorders have revealed broad alterations in protein signatures across multiple pathways, including the AhR signaling, glucose metabolism, redox regulation, mitochondrial integrity, and protein turnover mechanisms such as endocytosis, intracellular trafficking, and degradation. These convergent molecular disturbances underscore the central role of GABAergic interneurons in the pathophysiology of SCZ and mood disorders [101].

Tryptamine, an endogenous ligand, activates AhR and inhibits the expression of CYP1A and CYP2A enzymes. Deregulated tryptamine reflects serotonin imbalance and affects AhR functions. Excess tryptamine resulting from hypoxia activates HIF1, promoting AhR signaling and monoamine oxidase (MAO) activation across a wide range of neuropsychiatric disorders, including Alzheimer’s disease (AD), Parkinson’s disease (PD), autism, and SCZ [102].

**Figure 3:**
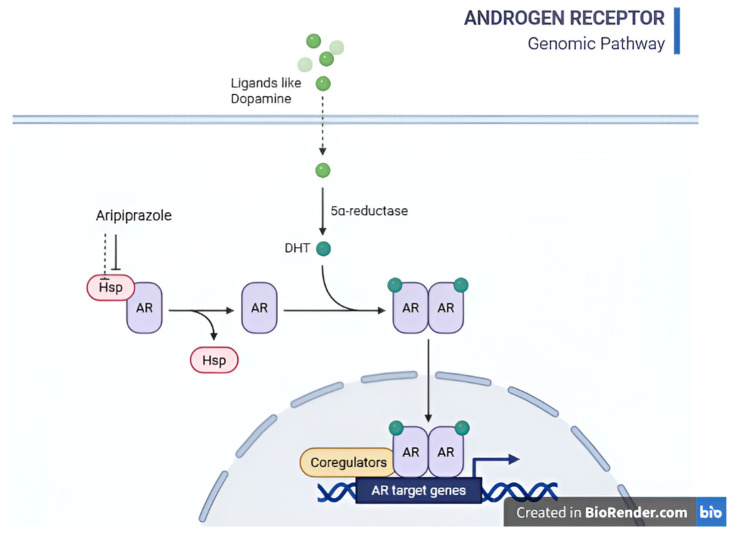
Schematic representation of AhR signaling pathway. In its dormant state, AhR resides in the cytoplasm bound to its chaperone HSP90. Upon activation by endogenous or exogenous ligands, AhR dissociates from HSP90, translocates to the nucleus, binds to DNA response elements, and promotes transcription of target genes involved in cellular aging and immune modulation. Dopamine and serotonin stimulate AhR to modulate the aging process. Aripiprazole blocks AhR dissociation from HSP90, potentially locking senescence.

Figure 3 illustrates the pathway of AhR from the cytoplasm to the nucleus, promoting cell aging in response to various endogenous and exogenous ligands.

### 6.2. RXR

RXR (Retinoic X receptor) is a nuclear receptor that forms heterodimers with multiple other nuclear receptors, including retinoic acid receptors, thyroid hormone receptors, and vitamin D receptors. RXR signaling plays a critical role in neuronal differentiation, synaptic plasticity, and brain development.

The schizophrenic brain demonstrates increased expression of retinoic acid-induced 1 (RAI1), which is transcribed to a nuclear TF essential for maintaining neuronal population stability [103]. Disrupted retinoic acid signaling in knockout animal models corresponds to the defective development of the prefrontal cortex’s mediodorsal connections with the thalamus, consistent with a neurodevelopmental contribution to SCZ pathology [104].

Microdeletion of the RAI1 gene is associated with a mosaic phenotype that induces a rare congenital syndrome characterized by craniofacial and skeletal deformities. A patient with this syndrome suffers from otolaryngeal and neuropsychiatric manifestations, including juvenile SCZ. Other findings include sleep disturbance, headache, and depression [105].

**Figure 4:**
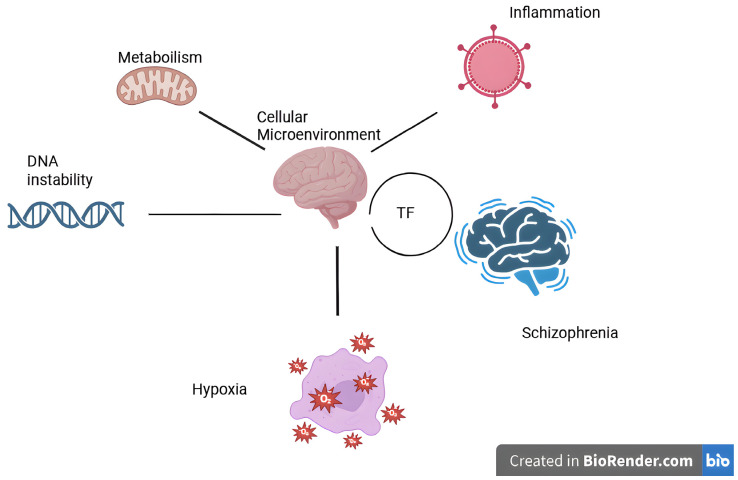
Schematic representation of TF interactions within the brain microenvironment. In SCZ, the cellular microenvironment is characterized by DNA instability, metabolic imbalance, inflammation, and hypoxia, all of which converge to promote disease pathogenesis through coordinated TF networks.

Figure 4 proposes that SCZ pathology is represented by a defective cellular microenvironment. By analyzing the different groups of TFs related to SCZ, it could be speculated that SCZ is a developmental, hypoxic, nuclear, and genomic disorder with a complex transcriptomic signature.

### 6.3. Other Nuclear Receptors

Table 1 summarizes a number of nuclear factors involved in the pathogenesis of SCZ. Many nuclear TFs are involved in the pathology of SCZ, raising questions about a major insult in the neuron nucleus.

**Table 1:** Additional nuclear receptors implicated in SCZ.

Nuclear Receptor	Potential Role
DAX1 like receptors	No major direct role
Thyroid Hormone receptors	Iodothyronine deiodinase 2 rs225014 is associated with a high risk of SCZ. Low thyroxin levels are related to hyper-sensitive dopamine receptors [106].
Peroxisome receptor alpha	SCZ pathology is related to PPARα-regulated transcriptomic signature and modulation of synapse functioning. Hence, PPARα can serve as a potential drug target for SCZ [107].
Peroxisome receptor Beta	No major direct role
Liver receptor X like receptors	No major Direct role
Vitamin D like receptors	Polymorphisms are associated with obesity in schizophrenic candidates [108].
Testicular receptors	No major Direct role
Tailless receptors	No Major Direct Role
COUP TF like receptors	No major Direct Role
Estrogen receptors	Estrogen signaling plays a role in cognition and neuroprotection as it mediates multiple pathways, including dopaminergic, serotonergic, and glutamatergic systems [109].
Hepatocyte nuclear factor 4	No Major Direct role
Estrogen-related receptor	No studies
Androgen Receptor	The relation between serum testosterone and ventral striatum pathology has been documented in SCZ. Midbrain dopamine neurons expressed a positive reaction to the androgen immune stain [110].
Mineralocorticoid and Glucocorticoid Receptors	Abnormal prefrontal cortex function and the hypothalamic-pituitary-adrenal (HPA) axis were altered and correlated with both glucocorticoid and mineralocorticoid receptor alterations in patients with affective disorders and SCZ [111]
Progesterone receptors	The process of pregnancy and delivery may be associated with postpartum psychosis. Synthetic neurosteroid has been applied to treat psychiatric disorders like SCZ [112].
Nerve Growth Factor 1B	No major Direct Role
Nuclear receptor related 1	No major Direct role
Neuron-derived orphan receptor 1, 2, 3	The expression levels of NR4A1, NR4A2, and NR4A3 were decreased following lifetime treatment with antipsychotics. These findings suggest the role of the orphan and retinoid nuclear receptors in the cortical dysfunction related to SCZ [113].
Steroidogenic factor 1	Related to neurodegenerative disorders like Huntington’s disease [114].
Liver receptor homolog-1	It plays a role in the liver-brain axis; however, liver nuclear transcription factors do not have enough evidence to be a risk factor for SCZ [115].
Germ cell nuclear factor (GCNF)	It has a role in embryogenesis and germ cell differentiation. It is expected to promote neurogenesis, ref. [116] but no evidence of a direct relation to SCZ. More research is needed.

## 7. Miscellaneous TFs Involved in SCZ

Table 2 presents a glossary of TFs implicated in the development of SCZ through various mechanisms. These TFs are associated with multiple aspects of neuronal function and the epigenetic regulation of the transcriptome linked to SCZ. Table 3 summarizes the common TFs with their common mechanisms and the evidence of their roles.

## 8. Integrated Insights and Therapeutic Implications

This review has implicated more than 40 TFs in the SCZ pathogenesis. Collectively, the evidence suggests that SCZ can be conceptualized as a disorder involving hypoxia, metabolic dysregulation, nuclear dysfunction, and disturbed immune responses. It is hypothesized that the schizophrenic brains exhibit a disrupted cellular microenvironment [133] analogous to the tumor microenvironment [134], with TFs playing diverse and interconnected roles.

The relationship between cancer and SCZ requires further exploration. Studies have demonstrated a decreased risk of breast cancer in patients with SCZ [135], while another study from Finland reported a high incidence of breast cancer in schizophrenic women [136]. These conflicting findings highlight the need for larger, well-controlled epidemiological studies. Notably, pimozide, a typical antipsychotic, possesses the ability to reprogram glucose metabolism in breast cancer cells by increasing p53 translation [137].

Dysfunctional activation of the AhR, triggered by toxins from gut microbiota or environmental sources, promotes premature cellular and neuronal aging, a hallmark of SCZ. Early brain senescence induces secondary changes, including mitochondrial impairment, gray matter reduction, inhibited gamma oscillations, and a metabolic shift toward lactate production and lactylation as a compensatory mechanism [138]. The current review suggests a role of AhR antagonists, and potentially agonists, in modulating the cellular microenvironment of the diseased brain [139].

Retinoic acid receptors bind to phospholipases A2, C, and D in the nuclear membrane, playing an important role in the redeployment of arachidonic acid in neuronal membranes during differentiation and growth. Abnormal retinoid metabolism may correspond to downstream transcriptional regulation of phospholipase A2-mediated signal transduction in SCZ and Alzheimer’s disease (AD). There is a need to develop new retinoid analogs with reduced toxicity that can safely cross the blood–brain barrier as a novel treatment of psychiatric and neurological disorders [140].

Bexarotene, a chemotherapy agent, activates retinoic acid receptors, modulates target gene expression, and induces dampening of the psychotic symptoms in patients with SCZ or schizoaffective disorders [141,142].

## 9. Challenges in Targeting TFs for SCZ Therapy

Targeting TFs in SCZ presents substantial challenges. As discussed throughout this review, a wide array of TFs is involved in the pathogenesis of psychotic disorders. In general, TFs lack defined binding pockets for small-molecule interaction, making them difficult to target directly [143].

Moreover, TFs exhibit dynamic three-dimensional structures, forming what has been described as “protein clouds” that respond to heterogeneous and potentially complex structured ligands. These findings may shift the therapeutic rationale towards protein-directed targeting strategies [144]. Furthermore, TF binds to DNA through a large, flat, positively charged surface, which complicates the design of classical small-molecular drugs [145].

An additional challenge is that TFs interact with numerous co-activators or co-repressors, increasing the risk of off-target effects [146]. Although TFs have been successfully targeted in other disease contexts, such as degenerative disorders, cancers, and genetic diseases, the drug delivery of synthetic TFs remains a significant obstacle. Advances in nanoparticle technology are under extensive investigation to enable effective TF-targeted therapies [147].

Experimental research has shown that drugs such as thalidomide and related immunomodulatory agents can recruit cereblon (CRBN) to the CRBN–Cullin-Ring E3 ubiquitin ligase complex, promoting targeted degradation of specific TFs. This indirect mechanism of modulating TF abundance highlights the potential of CRBN-based therapeutics to influence protein stability and cellular signaling networks [148]. Despite its effectiveness in oncology, thalidomide has not shown benefit in psychosis; indeed, its use in ulcerative colitis has been linked to psychotic symptoms [149]. This divergence reflects fundamental differences in TF biology between cancer and SCZ rather than a failure of the therapeutic strategy itself.

Experimental studies have utilized CRISPR technology and engineered (designer) TFs to improve cognitive function in animal models of SCZ. However, these approaches remain largely experimental and have not yet been widely translated into human clinical applications. Compared with fields such as cancer therapy, where TF targeting is far more advanced, therapeutic applications in SCZ remain relatively underdeveloped in terms of precision drug-targeting strategies [150,151].

Given the design of this review, it is limited in its quantitative or methodological quality evaluation of synthesized evidence, subjecting it to publication bias. Furthermore, the inclusion of only English-language studies might have overlooked other critical evidence published in various other languages. Future research requires high-throughput, single-cell transcriptomic studies and longitudinal analyses to further explore TF networks in SCZ.

## 10. Conclusions

Schizophrenia (SCZ) is a devastating psychological disease with a progressive clinical course. This review has implicated a vast number of TFs in the pathogenesis of SCZ or in the risk of developing psychosis. The current work has categorized these TFs into developmental (progenitor), stem cell, metabolic reprogramming, and nuclear groups. The nature of these protein-DNA-interacting agents reflects profound genomic instability, immune dysregulation, and metabolic disturbance underlying SCZ. Despite the challenging, often termed “undruggable” nature of TFs, these complex proteins continue to offer promise for understanding SCZ pathogenesis and identifying novel therapeutic targets. Promising avenues include drugs that modulate aryl hydrocarbon receptor (AhR) or retinoid X receptor (RXR) signaling pathways. Antagonists of AhR may slow the aging process associated with SCZ progression and modulate immune responses. Activators of RXR may support blood–brain barrier integrity, thereby limiting the brain exposure to different toxins. SCZ exhibits a unique cellular microenvironment in which the immune system is heavily involved. Advances in immunotherapy may therefore play a significant role in future treatment strategies.

## Figures and Tables

**Table 2:** Miscellaneous TFs implicated in SCZ.

Transcription Factor	Mechanism
NPAS3 (Neuronal PAS Domain Protein 3)	Alterations of Npas4 expression and protein unfolding correspond to SCZ [117].
NAPS4 (Neuronal PAS Domain 4)	Low expression of Npas4 in parvalbumin interneurons and the associated cognitive decline under neonatal NMDA receptor blockade correspond to SCZ [118].
SP (specific protein family)	SP4 is a risk factor for SCZ but may also act as a platform for signaling link in the regulation of many other SCZ-risk signature [119].
Lhx6 (LIM home box protein)	Defective transcription factor Lhx6, which regulates parvalbumin and somatostatin neuron development, is correlated with subsequent GAD67 dysfunction in SCZ. GAD67 (glutamate decarboxylase 67) knockout animals expressed low Lhx6 expression with the manifestation of SCZ [120].
EZH1 (Enhancer of zeste homolog 1)	EZH1 was abnormally expressed in SCZ. It is affected by antipsychotic medicines; studies demonstrated high transcription of miR-132 and EZH1 in the frontal cortex of psychotic mouse models [121].
EZH2 (Enhancer of zeste homolog 2)	RNA-seq data obtained from PD_NGSAtlas, which reflects the record for epigenomic and transcriptomic data for psychiatric disorders, revealed a significant increase in EZH2 expression in the anterior cingulate cortex of schizophrenic patients. Studies suggest that EZH2 may contribute to the increased risk of SCZ through developmental disruption or increased expression in the mature brain [122]. It modifies histone methylation, suggesting epigenetic control [123].
C-Jun is a protein that forms part of the Activator Protein-1 (AP-1) TF complex	Chronic animal model of psychosis by MK-801 repeated treatment demonstrated decreased phosphorylation and protein level of c-Jun in the rat frontal forebrain [124]. However, post-mortem schizophrenic brains revealed increased expression of c-Jun in the vermis of the cerebellum [125].
MEF2C (Myocyte Enhancer Factor 2C)	It has an essential role in neurogenesis and synapse maturation. Studies raised questions about the role of MEF2c in the risk of the development of neuropsychiatric disorders, including SCZ. This transcription factor modulates a group of genes involved in a wide range of biological mechanisms, including neuron mitosis, differentiation, and development. Furthermore, it is involved in mitochondrial function and energy metabolism [126].
(MYT1L) The myelin TF-1-like	Polymorphisms of MYT1l have been associated with the risk of SCZ in Chinese studies [127], and Australian results [128].
TEAD1:TEA domain TF 1	An experimental polymorphism genetic study indicated that the human TEAD1 gene has an increased risk related to SCZ in the Chinese Han samples [129].
The ZEB2 protein (Zinc Finger E-box binding 2).	Chinese studies concluded a potential risk of SCZ with that protein [130].
PRDM14	PRDM14 promoter is hypermethylated in a large-scale SCZ population [131].
NEUROG1	The EGR1-miR-30a-5p-NEUROD1 pathway is a biomarker in patients suffering from psychosis and possibly a target to treat SCZ [132].

**Table 3:** Common SCZ-related TFs along with evidence and proposed mechanism.

Transcription Factor	Genomic Location	SCZ Evidence	Proposed Mechanism
**Progenitor Cell TF**			
TCF4	18q21.2	Human and animal	Risk factor for cognitive disability, needs further research [11]
POU3F2	6q16.1	Human	Undetermined
NKX2	14q13.3	Human and Animal	Gene, Calcium dynamics, and immune [17]
**Stem Cell TF**			
MYC	8q24.21	Human	Modulate non-coding RNA [36]
SOX2	3q26.33	Human and animal	GABAergic interneurons, glutamatergic projection neurons [37]
ASCL1	12q23.2	Human	Neuroplasticity [5]
REST	4q12	Human	Essential for embryogenesis and neurogenesis [41]
**Metabolic reprogrammed**			
HIF-1α	14q23.2	Human	Hypoxia drives a plethora of growth factors [54]
SREBPs	SREBP1 17p11.2 SREBP2 22q13	Human and animal	Myelin and cholesterol metabolism [55,56]
STAT1	2q32.2	Human	Immune modulation [60]
SOX9	q24.3	Human and animal	Epigenetic control of the neural crest [67]
**Nuclear receptors**			
NR2E1	6q21	Animal	Essential for neurogenesis [43]

## Data Availability

No new data were created or analyzed in this study. Data sharing is not applicable to this article.

## List of Abbreviations

SCZ	Schizophrenia
TCF4	Transcription Factor 4
SOX	*SRY*-related HMG-box
Wnt	Wingless-related integration site
VIP	Vasoactive intestinal peptide
CST	Cortistatin
POU3F2	POU domain, class 3, transcription factor 2
NPCs	human neural progenitor cells
TRIM8	Tripartite motif containing 8
NKX2-1	NK2 homeobox 1
EGR3	Early Growth Response 3
GWAS	Genome-wide association studies
TF	Transcription factor
miRNA	Micro RNA
ARC	Activity-regulated Cytoskeleton Associated
OCT4	Octamer-binding transcription factor 4.
cMyc	cellular “myelocytomatosis”
Klf4	Kruppel-like factor 4
SOX2 OT	SOX2 overlapping transcript
Ascl1	Achaete-scute homolog 1
bHLH	basic-helix-loop-helix
scRNA-seq	Single-cell RNA sequencing
PHOX2B	Paired-Like Homeobox 2B
REST	RE1-silencing transcription factor
NRSF	Neuron-restrictive silencing factor
PET	Positron emission tomography
MRI	Magnetic resonance imaging
NMDA	N-methyl-D-aspartate
CA3	Third region of the Cornu Ammonis (CA)
NR2E1	The nuclear receptor 2E1
HIF1	Hypoxia-Inducible Factor 1
SREBPs	Sterol Regulatory Element-binding Proteins
POU1F1	POU Domain Class 1 Homeobox 1
STATs	Signal Transducer and Activator of Transcription
SOX9	SRY-related HMG box gene 9
NRF1	Nuclear factor erythroid-derived 2-related factor 1
NRF2	Nuclear factor erythroid-derived 2-related factor 2
PBX3	pre-B-cell leukemia transcription factor 3
MIF	Macrophage migration inhibitory factor
SNP	Single-nucleotide polymorphisms
AKT1	Serine/threonine kinase 1
BDNF	brain-derived neurotrophic factor
CAPON	carboxy-terminal PDZ ligand of neuronal nitric oxide synthase.
CCKAR	cholecystokinin A receptor
CHRNA7	cholinergic receptor nicotinic alpha 7 subunit
CNR1	cannabinoid receptor 1
COMT	Catechol-O-methyltransferase
DTNBP1	dystrobrevin-binding protein 1
GAD1	glutamate decarboxylase 1
GRM3	Glutamate Metabotropic Receptor 3
IL10	Interleukin-10
MLC1	myosin light chain 1 protein
NOTCH4	Neurogenic Locus Notch Homolog Protein 4
NRG1	neuregulin 1
NR4A2/NURR1	The nuclear receptor 4A2/nuclear receptor related 1 protein
PRODH	proline dehydrogenase
RELN	Reelin
RGS4	Regulator of G-protein signaling 4
RTN4/NOGO	Reticulon 4: a gene that encodes a family of proteins known as Nogo
TNF	Tumor Necrosis Factor
JAK	Janus kinase
RAC1	Ras-related C3 botulinum toxin substrate 1,
NR4A2	nuclear receptor subfamily 4 group A member 2)
LC1A3	solute carrier family 1 member 3
AQP4	Aquaporin-4
GJA1	gap junction protein alpha 1
ALDH1L1	10-formyltetrahydrofolate dehydrogenase
SLC4A4	10-formyltetrahydrofolate dehydrogenase
EGR1	Early growth response protein 1
NOTCH2	Neurogenic locus notch homolog protein 2
PVALB	gene symbol for the parvalbumin protein
ID4	Inhibitor of DNA binding 4 or Inhibitor of Differentiation 4
ABCG2	ATP-binding cassette subfamily G member 2
METTL7A	methyltransferase-like protein 7A
F3	Coagulation Factor III
EMX2	empty spiracles homeobox 2
PGC-1α	Peroxisome proliferator-activated receptor gamma coactivator 1-alph
Syt2	Synaptotagmin 2
Cplx1	Complexin 1
TINK	Twinkle mtDNA helicase
TRAF2	TNF receptor-associated factor 2
NCK	Non-catalytic region of tyrosine kinase
Nfe212	Nuclear Factor Erythroid 2-Related Factor 2
HO-1	heme oxygenase-1
Nqo1	NAD(P)H quinone oxidoreductase 1
Trx	TRanscription and EXport
GABA	gamma-aminobutyric acid.
KEAP1	Kelch-like ECH-associated protein 1
p53	tumor protein p53
FOXO	Forkhead box, class O
DAF-16	the name of a gene and the protein it encodes in the nematode *C. elegans*
ATF4	Activating transcription factor 4
iPSC	Induced pluripotent stem cells
DSC1	Disrupted in Schizophrenia 1
(NF-κB)	Nuclear factor-kappaB
IL17	Interleukin 17
TNF	Tumor necrosis alpha
GRIN1	Glutamate Receptor Ionotropic, N-Methyl D-Aspartate 1
AhR	Aryl hydrocarbon receptor
CYPA1	Cytochrome P450, Family 1, Subfamily A
CYP2A	Cytochrome P450, Family 2, Subfamily A
RXR	Retinoid X Receptor
RAI1	retinoic acid induced 1
DAX1	Dosage sensitive sex reversal, Adrenal hypoplasia congenita critical region on the X chromosome, gene 1
COUP TF	Chicken Ovalbumin Upstream Promoter-Transcription Factor
GCNF	Germ Cell Nuclear Factor
NPAS3	Neuronal PAS domain-containing protein 3
NPAS4	Neuronal PAS domain-containing protein 4
SP	Specificity Protein
Lhx6	LIM/homeobox protein
EZH1	Enhancer of zeste homolog 1
EZH2	Enhancer of zeste homolog 2
C-Jun	Transcription factor Jun
MEF2C	myocyte enhancer factor 2C
MYT1L	Myelin Transcription Factor 1-Like
TEAD1:TEA	TEA domain transcription factor 1
ZEB2	Zinc finger E-box-binding homeobox 2
PRDM14	PR domain-containing protein 14
NEUROG1	Neurogenin 1
CRBN E3	cereblon E3 ubiquitin ligase complex
